# Genetic characterization of porcine circovirus type 2 in piglets from PMWS-affected and -negative farms in Thailand

**DOI:** 10.1186/1743-422X-8-88

**Published:** 2011-02-28

**Authors:** Tippawan Jantafong, Alongkot Boonsoongnern, Pariwat Poolperm, Kitcha Urairong, Chalermpol Lekcharoensuk, Porntippa Lekcharoensuk

**Affiliations:** 1 Department of Microbiology and Immunology, Faculty of Veterinary Medicine, Kasetsart University, Thailand; 2 Department of Farm Resources and Production Medicine, Faculty of Veterinary Medicine, Kasetsart University, Thailand; 3 Department of Companion Animals Clinical Sciences, Faculty of Veterinary Medicine, Kasetsart University, Thailand

## Abstract

Porcine circovirus type 2 (PCV2) is the major swine pathogen associated with Porcine circovirus associated disease (PCVAD) including post-weaning multisystemic wasting syndrome (PMWS). Currently, there are 4 subtypes of PCV2 (PCV2a, b, c and d) and some epidemiological evidences demonstrated that virulence of PCV2 may relate to its subtypes. Recently, PMWS was observed more frequently in swine farms in Thailand; however, the information regarding to PCV2 subtype involved was limited. Therefore, this study was aimed to determine the association between occurrence of PMWS and PCV2 subtypes as well as genetically characterize PCV2 in Thailand. PCV2 DNA was isolated from faecal swabs and whole blood of piglets from PMWS-affected and -negative farms. The full length ORF2 sequences were compared using multiple alignment. The results showed that PCV2 DNA was detected more frequently in PMWS-affected farms. The nucleotide identities of the ORF2 from 9 PCV2 isolates representing each PMWS-affected farm and one from the negative farm ranged from 92.4 to 99.5% suggesting that there is some genetic variation of PCV2 in Thai swine. The 10 PCV2 isolates were classified into 2 clusters, in which the 7 isolates from PMWS-positive farms were in PCV2b cluster 1 A/B. The remaining isolates were separated in the new subtype called PCV2e. The results suggest the presence of new PCV2 subtypes in addition to PCV2a and PCV2b in Asian swine population. However, correlation between subtypes and virulence of PCV2 infection is not conclusive due to limited number of the PCV2 sequences from PMWS negative farms.

## Findings

Postweaning multisystemic wasting syndrome (PMWS) is a major disease of swine, caused by porcine circovirus type 2 (PCV2). The major clinical signs of PMWS are wasting, paleness of the skin, enlargement of the lymph nodes, respiratory distress, and occasional diarrhea as well as icterus [1,2]. PCV2 has a significant impact on the pig industry worldwide [3]. PCV2 is a small non-enveloped DNA virus which contains a single-stranded circular genome of 1.7 kb [4,5]. The PCV2 genome has three major open reading frames (ORFs), which encode replication-associated proteins (ORF1) [6], viral capsid protein (ORF2) [7], and apoptotic protein (ORF3) [8]. The ORF2 is an essential determinant for the genetic typing of PCV2 isolates, since the capsid gene has more variability than the other ORFs [9]. Phylogenetic analysis demonstrated two major subtypes of PCV2, which are PCV2a (Group2) and PCV2b (Group 1). Each subtype was classified into different clusters, 1A to 1C for the subtype PCV2b, and 2A to 2E for the subtype PCV2a [9]. Furthermore, a third subtype (PCV2c), found only in the Danish pigs, was described [10]. A novel subtype, PCV2d, which is dominant in Chinese pigs, was recently reported [11]. A number of studies suggested that PCV2b is more virulent than PCV2a and predominant in PMWS-affected herds [12,13]. However, an experimental inoculation of pigs with PCV2a and PCV2b demonstrated no divergence in the virulence of both subtypes [14]. Thus, no association of the PCV2 subtype designations, the disease status, or the geographical distribution appears to be evident.

Since PMWS was first observed in Canada in 1991 [1], the prevalence of PCV2 and the occurrence of PMWS have been worldwide reported [15-25]. The first case of PMWS in 7 to 9 week-old pigs was described in Thailand in 1998 [26]; however, a retrospective study suggested that the PCV2 infection had been previously detected in 1993 [21]. Although the infection has been widespread in Thailand since 1998, genetic information of PCV2 is limited with only one sequence (AY864814) deposited in the GenBank databases. Therefore, the aim of this study was to genetically characterize the PCV2 isolates from Thai swine and to determine the prevalence of the PCV2 infection in piglet from both positively and negatively PMWS affected farms.

**Figure 1:**
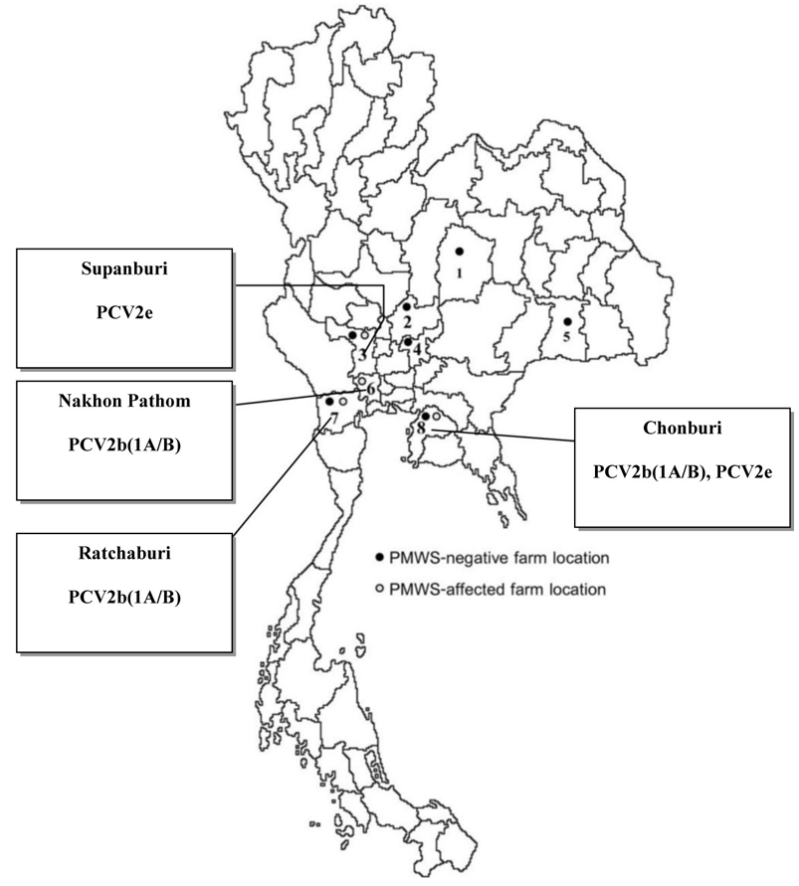
**Locations of the pig farms participating in this study**. Black sphere (●) represents PMWS-negative farms and hollow sphere (○) represents PMWS-affected farms. The farms analyzed are located in the eastern, north-eastern, western, and central parts of Thailand. The numbers represent the provinces in which the farms are located; 1 = Chaiyaphum, 2 = Lopburi, 3 = Suphanburi, 4 = Saraburi, 5 = Buriram, 6 = Nakhon Pathom, 7 = Ratchaburi, and 8 = Chonburi.

Based on a preliminary PCV2 survey with PCR, the prevalence of the PCV2 infection in Thailand was ~10%. Thus, 14 PMWS-negative farms and 11 PMWS-affected farms were included in this study. The farms were located in the eastern (Chonburi province), north eastern (Buriram and Chiyaphum provinces), western (Ratchaburi province), and central (Lopburi, Nakhon Pathom, Saraburi, and Supanburi provinces) parts of Thailand. Fourteen PMWS-negative farms located in Chonburi, Lopburi, Saraburi, Suphanburi, Ratchaburi, Chaiyaphum, and Buriram provinces, along with eleven PMWS-affected farms located in Chonburi, Suphanburi, Ratchaburi, and Nakorn Pathom provinces were selected (Figure 1). The pigs from the PMWS affected farms were selected in accordance with the following clinical presentations; (1) clinical signs characterized by wasting, paleness of the skin, and respiratory distress; (2) lymph node enlargement and (3) a > 5 to 10% production loss in the nursery and fattening groups. In addition, the sick pigs from these farms were submitted to the Kasetsart University Diagnosis Laboratory at Faculty of Veterinary Medicine, Kampaengsean Campus, for necropsy before the commencement of the study. The PMWS negative farms were selected as to agree with the following characteristics: (1) farms absent of a PMWS history, (2) farms absent of a clinical presentation of PMWS, and (3) farms with production losses of less than 4%. The samples were collected prior to the implementation of the PCV2-vaccintion in Thailand. A total of 140 pigs were collected in a period from July 2007 to March 2008. Whole blood and faecal swabs were randomly collected from five to ten piglets at ages of 5-10 weeks from the PMWS-negative farms (n = 70) and the PMWS-affected farms (n = 70). Whole blood samples in Ca-EDTA tubes were centrifuged, and the plasma was collected and subsequently frozen at -80°C until used. The faecal swabs were stored in a virus transport medium, vortexed and clarified by means of centrifugation with subsequent storage of the supernatants at -80°C.

Total DNA was extracted from the plasma and the suspension of faecal swabs with the application of a Nucleospin^® ^Blood kit (Macherey-Nagel, Germany) in accordance with the manufacturer's instructions. The isolated DNA was amplified by means of PCR with the PCV2 specific primers [27]. Descriptive statistics, with the employment of the NCSS 2007 program, were utilized to assess the overall data quality. Associations between the percentages of the PCV2 positive in PMWS-affected and PMWS-negative farms and the manifestation of PCV2 DNA in faecal swabs as well as in the whole blood samples were analyzed with the chi-square test. A *P *value of less than 0.05 was regarded as statistically significant. A positive PCR sample was selected from each farm for sequence analysis.

The results of the PCV2 DNA from faecal swabs and whole blood samples detected with PCR are shown in table 1. PCV2 was found in 52 of 140 pigs (37.14%) from both farm categories. A percentage of 7.14 of all faecal swabs collected form piglets form PMWS-negative farms was determined, while those from PMWS-affected farms attained 50%. The PCV2 DNA was not detected in the whole blood samples from the PMWS-negative farms, while 57.14% of pigs from the PMWS-affected farms showed PCV2 viremia. PCV2 DNA detection in the blood or faecal samples from the PMWS-affected farms were found not to be significantly different (χ^2 ^= 3.83, *P *> 0.05). However, higher percentages of PCV2 were detected in either blood or faecal samples of pigs from the PMWS-affected farms (67.14%), in comparison to those from the non-PMWS farms (7.14%). The PCV2 DNA in both specimens was found in 27 samples; all of which were obtained from the PMWS-positive farms. Furthermore, the appearance of the PCV2 DNA in the PMWS-affected farms in Thailand is age-dependent. The PCV2-positive ratios found in 5, 6, and 8-10 week-old piglets were 43.3 (n = 30), 80 (n = 25), and 100% (n = 15; 5 pigs from each age group), respectively. Overall, the detection of the PCV2 DNA was significantly related to the PMWS (χ^2 ^= 53.96, *P *< 0.01). These findings are in agreement with the recent studies as well as previous field observations [28,29].

**Table 1:** PCR amplification of PCV2 DNA from whole blood and faecal swab samples

Samples	PCR results	Number of pigs in farms		Total
			
		PMWS -	PMWS +	
Faecal swabs	Negative	65	35	100
	Positive	5 (7.14%)	35 (50%)	40

	Total	70	70	140

Whole blood	Negative	70	30	100
	Positive	0 (0%)	40 (57.14%)	40

	Total	70	70	140

In addition, the full length of the ORF2 of PCV2 was amplified with the PCV2-ORF2 specific primers [7,30]. The PCR products were submitted for sequencing. The 10 ORF2 sequences (nine from PMWS-affected pigs and one from a negative pig) from the present study and 82 ORF2 sequences from GenBank were analyzed with the application of the ClustalW method provided in the DNASTAR software. The ORF2 gene sequence of PCV1 (AY699796) was used as the out-group. The phylogenetic tree was built by means of the neighbour-joining (NJ) method in MEGA 4 program with 1000 bootstrap replicates.

The results demonstrated the nucleotide identities of those 10 isolates to range between 92.4-99.5% (Table 2). A PCV2 isolate from a PMWS-negative farm and two PCV2 isolates from PMWS-affected farms shared 99.1-99.5% nucleotide identities, which suggests an absence of any significant divergences in the PCV2 subtype from PMWS-affected and -negative farms [31-33]. Nucleotides 262-267 and amino acids 88-89 of ORF2 were compared and classified as previously described [34], in which the consensus "CCCCGC", "CCCCTC", and "AAAATC" are the signatures for the PCV2b cluster 1A/1B, 1C, and PCV2a, respectively. The multiple alignment of the ORF2 gene revealed that all 10 PCV2 isolates contain the PCV2b signature (Table 2). The three PCV2 isolates, THKUF49, THKUL16, and THKUB98 have a cluster 1C signature, while the other seven PCV2 isolates from the PMWS-affected farms possess a cluster 1A/B signature.

**Table 2:** Classification of PCV2 strains based on nucleotides 262-267 of the ORF2 sequences

Isolates	Nucleotide sequence (262-267)	Subtype	Cluster	Origin	% identity^b^	Accession number
**AF201311**^a^	**CCCCGC**	**PCV2b**	**1A**	**France**	**100%**	AF201311
**AY484407**^a^	**CCCCGC**	**PCV2b**	**1B**	**Netherlands**	**100%**	AY484407
THKUL74	CCCCGC	PCV2b	1A/B	Nakhon Pathom	98.9/99.8%^c^	HQ735207
THKUB115	CCCCGC	PCV2b	1A/B	Ratchaburi	98.3/99.2% ^c^	HQ735204
THKUB132	CCCCGC	PCV2b	1A/B	Ratchaburi	98.6/99.5% ^c^	HQ735205
THKUB95	CCCCGC	PCV2b	1A/B	Chonburi	97.2/98.1% ^c^	HQ735202
THKUB85	CCCCGC	PCV2b	1A/B	Chonburi	98.6/99.5% ^c^	HQ735201
THKUSP	CCCCGC	PCV2b	1A/B	Ratchaburi	98.9/99.8% ^c^	HQ735200
THKUB136	CCCCGC	PCV2b	1A/B	Nakhon Pathom	99.1/100%^c^	HQ735206
**AY864814**^a^	**CCCCTC**	**PCV2b**	**1C**	**Thailand**	**100%**	AY864814
THKUF49	CCCCTC	PCV2b	1C	Chonburi	98.6%	HQ701666
THKUL16	CCCCTC	PCV2b	1C	Chonburi	98.9%	HQ701665
THKUB98	CCCCTC	PCV2b	1C	Suphanburi	99.1%	HQ735203
**AF201307**^a^	**AAAATC**	**PCV2a**	**2**	**Germany**	**100%**	AF201307

^a^Boldface letters represent reference sequences.

^b^Comparison of the isolates in the first column and the reference nucleotide sequences in the identical group.

^c^Comparison of each individual Thai isolate and the reference sequences in cluster 1A (AF201311)/1B (AY4884407).

**Figure 2:**
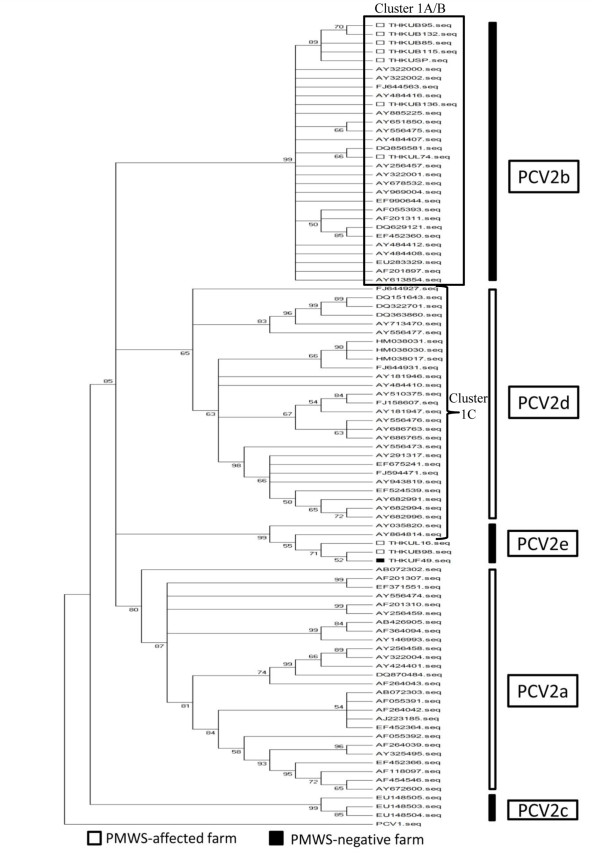
**Phylogenetic analysis of the complete ORF2 sequence of the PCV2 isolates in this study**. The tree was constructed from the 82 ORF2 sequences deposited in GenBank and 10 Thai isolates in the present study with the application of the MEGA 4 program. The ORF2 of PCV1 was used as an out-group. Note that clusters 1 A/B and C indicate the previous classification [9].

The phylogenetic tree revealed that the sequences were segregated into five major subtypes, PCV2a, PCV2b, PCV2c, PCV2d, and a new Thai subtype referred to as PCV2e (Figure 2). In comparison to a previous study [9], the PCV2b group in figure 2 is formerly classified as PCV2b (group1) cluster 1A/B, while those in PCV2d and PCV2e groups were previously classified as PCV2b cluster 1C. In the present study, the seven PCV2 isolates from the PMWS-affected farms were classified in PCV2b (cluster 1A/B). The other three isolates (THKUB98, THKUL16, and THKUF49) with the cluster 1C signature are more distant and form a new cluster together with the former Thai isolate (AY864814) and a Chinese isolate (AY035820) (Figure 2). The sequence distances between ORF2 of the later cluster and those in the other clusters were found to exceed 3.5%, which qualifies for the characterization of a new subtype [12] referred to as PCV2e, in keeping with the recently determined new Chinese subtype PCV2d [11]. The Chinese PCV2 (AY035820) isolated in 2001 from PMWS pig in eastern China and the Thai isolates (AY864814) reported in 2004 coincidentally appear to possess the PCV2b cluster 1C signature. The Chinese PCV2 is therefore potentially the ancestor of the Thai PCV2 found in 2004, which in turn was the origin of the three 2007 PCV2 isolated in the present study.

In conclusion, the present study constitutes the first genetic analysis of the Thai PCV2 isolates, which determines the correlation of the occurrence of PMWS and the PCV2 subtypes in the Thailand. This study also demonstrated that PCV2 infection associates with the occurrence of PMWS. In addition, the circulation of the PCV2 strains appears to be currently increasing in the Thai swine population. Two subtypes, PCV2e and PCV2b (1A/B), are at present existent in the Thai swine population, where PCV2b appears to be dominant. Phylogenetic analysis of the recent PCV2 strains from the present study has confirmed the existence of more PCV2 subtypes in addition to PCV2a and PCV2b. As new genetic clusters of PCV2 have continually emerged, the classification of the PCV2 subtypes and clusters needs to be reconsidered by the PCV2 taxonomists as to set standard criteria for the PCV2 genetic characterization.

## Competing interests

The authors declare that they have no competing interests.

## Authors' contributions

TJ performed both the laboratory experiments and the phylogenetic analysis as well as the draft of the manuscript under the supervision of PL. AB, PD, and KU participated in the necropsy and sample collection. CL contributed to the study design and performed the statistical analysis. PL: designed the study and revised the manuscript. The final draft of the manuscript was seen and approved by all the authors.
